# Journey to the East: a focused review of online mental health research in East Asia

**DOI:** 10.3389/fpubh.2026.1841986

**Published:** 2026-10-12

**Authors:** Christine Linda Cook, Yun-Chung Tang, Honoka Fukuda, Juyeon Shin

**Affiliations:** 1 National Chengchi University, Taipei, Taiwan; 2 Center for Asia-Pacific Resilience and Innovation, Taipei, Taiwan

**Keywords:** China, East Asia (EA), Internet Gaming Disorder (IGD), Japan, mental health, online behavior, South Korea, Taiwan

## Abstract

**Introduction:**

Despite the population and connectivity of many countries in East Asia, its voice has not been fully explored in the ongoing global conversations around mental health and online behavior. To bring the research that currently exists to international attention while identifying areas where additional research can be done, the current systematic/focused review was conducted.

**Methods:**

Using the PRISMA methodology, we gathered over 1500 articles and ultimately reviewed 54 articles crossing multiple disciplines and languages (English, Korean, and Japanese).

**Results:**

The present work identifies key regional patterns in terms of topics covered and methodologies employed, noting that there is a major focus on addiction and general negative framing around online behavior. Broader research themes identified include Internet Gaming Disorder/Addiction (IGD), social media, internet, and smartphone addictions, clinical diagnoses and detection methodologies, as well as body and identity.

**Discussion:**

Mental health research in many parts of East Asia is negatively framed, irrespective of language of publication. Though cultural factors are likely to be at play, the present work would suggest that significant investment of time and personnel needs to be made in examining positive care using technology as opposed to focusing heavily on technology’s dangers.

## Introduction

An increasing number of studies from around the world suggest that, at the time of writing, we are experiencing a global mental health crisis [see (1–4)]. This has many potential causes: an overreliance on pharmaceuticals (1), systemic inequalities (3), and an uptick in diagnoses after the COVID-19 pandemic (2) have all been suggested in existing literature. Academic consensus has yet to be reached in any given region, let alone globally. However, within this collective search for both cause and solution, one major voice remains comparatively silent: the countries in East Asia. Despite being home to over an estimated 1.6 billion of the world’s population (5), the majority of the studies on mental health, though occasionally dealing with diasporic communities (see for an example), focus on WEIRD–Western, educated, industrialized, rich, and democratic (6)–populations like the United States or Australia. Grounded in the cultural-ecosocial systemic approach (7), mental health is a multi-scale process where neurobiological mechanisms interact dynamically with specific eco-social contexts. Cross-regional and cross-cultural comparisons are therefore crucial, as distinct social conditions and cultural meanings continuously reshape mind–body regulation and disease expression through circular causality.

East Asian countries have the potential to present a unique voice within the realm of mental health research as it fits four out of the five of Henrich et al.’ (6) aforementioned criteria. The Asian Economic Tigers–Singapore, Hong Kong, South Korea, Japan, and Taiwan–all boast impressive economies supported by a variety of industries, and over half are democratically-governed [see (8)]. According to Morris (9), these modern economic miracles were largely driven by the region’s focus on education: both technical and more traditional higher education. In short, the populations of East Asia are educated, industrialized, rich, and–for the most part–democratic; it is only not Western. By “Western,” Henrich et al. (6) appear to have meant “of the Western hemisphere”, although the term is not explicitly defined in the original paper. Indeed, van Pinxteren (10) asserts that Henrich et al. (6) were really referring to the United States, and that this population does not represent the Western world any better than the Western world represents the entire world. He argues that we should focus more on core societal values. By examining East Asia specifically Japan, South Korea, and Taiwan (hereafter, ‘the three East Asian countries’), the present work has the potential to isolate what impact being ‘Western’ has on mental health perspectives, all other major sample elements being similar to the current critical mass of existing literature. Given the core values shared across many of these East Asian countries–e.g., education and social harmony [see (9)]–the present work also expands beyond the WEIRD framework to look at shared cultural constructs more broadly across the region.

The aim of the present work is therefore to conduct a focused review of mental health research in three East Asian regions: Japan, South Korea, and the Chinese-speaking regions outside of the mainland (Taiwan and Hong Kong, primarily). Contrary to the majority of existing systematic or focused reviews, this study incorporates multiple languages of publication into its review: specifically, English, Traditional Chinese–the dominant writing system of Hong Kong and Taiwan (11), Japanese, and Korean. In this way, we cast a broader proverbial net in which to capture as many possible perspectives within the three East Asian regions’ context as possible, not just those that are either desirable or palatable to Western publications. Eligible articles from a variety of social science and scientific databases both international and specific to the relevant regions are thematically analyzed by the team to determine common methodologies and specific topics within the umbrella of mental health research conducted in this part of the world.

## Methodology

The study employed the latest PRISMA (Preferred Reporting Items for Systematic Reviews and Meta-Analyses) methodology released in 2020 to capture the current state of online mental health research in three East Asian regions’ online communities. The review was conducted between August and November 2025 and included publications in English, Japanese, Korean, and Traditional Chinese. All publications were reviewed by a multilingual research team composed of researchers who are native speakers of English, Japanese, Korean, and Mandarin. Predefined procedures and criteria were used to identify, screen, and select the literature, as detailed in the following sections.

### Information sources

The literature search was conducted across multiple databases. For English-language publications, Scopus, EBSCO, ACM, and IEEE were selected due to their coverage across various disciplines. ACM and IEEE were specifically included given the study’s focus on online communities. For Japanese, Korean, and Traditional Chinese publications, CiNii, KCI, and Airiti were used, respectively, as well-established local databases. The respective search date of each database and further information are presented in Table 1.

**Table 1:** Data regarding databases employed and subsequent results.

Database	Language	Search date	Raw results	Screened results
Scopus	English	12/11/2025	731	28
EBSCO	English	14/10/2025	258	3
	Japanese	14/10/2025	11	0
	Traditional Chinese	09/10/2025	4	0
IEEE	English	13/11/2025	121	0
CiNii	Japanese	11/12/2025	250	6
KCI	Korean	14/09/2025	328	17
Airiti	Traditional Chinese	09/10/2025	12	0

All languages were searched on the first three databases; if a language is not indicated in the table, it means that that search in that language produced no results in that database. One article from KCI was written in English, but otherwise, languages are database-corresponding.

Although this selection is as broad as possible given accessibility limitations, future work could expand on this by incorporating single-journal databases and/or paywalled databases that might require membership to access. This could potentially allow for more studies in the target languages aside from English who may publish in non-indexed journals or in association-specific venues. However, the present work does account for the majority of publicly-available, broadly accessible articles on mental health in the three East Asian regions.

### Keyword generation

To generate keywords for this project, our end goal was vastness and exploration. As mentioned in the introduction, mental health in East Asia is neither as popular a research topic nor as established a field as in many Western nations. The authorial team therefore had several meetings over the course of roughly 1 month in which keywords were collectively brainstormed, discussed, and either included or rejected based on consensus regarding its reflection of (a) the three East Asian regions’ research, (b) online phenomena associated with mental health (positive or negative), and/or (c) techniques or strategies associated with improving (i.e., therapy, support group, coping strategies) or declining (i.e., cyberbullying, trolling, suicidal ideation) mental health.

At the outset, our intention was to focus specifically on online communities, as this is the target of the special collection. However, preliminary searches using Boolean text including words like “online community” or “communities” as a must-include term produced minimal results, even in English. We therefore decided to remove this and broadly examine research on mental health in Asia, with the component of online interaction as a preferred option. This resulted in the full list of keywords presented in Boolean syntax in Appendix A which we then axially divided into five broad categories: region, internet community, mental health, behaviours, and mixed (pertaining to multiple of these broader categories).

### Search strategies

For English searches, the syntax was written as follows: (Region) AND (Internet community) AND (Mental health OR Mixed) AND (Behaviours). Regarding non-English searches (i.e., Traditional Chinese/Mandarin, Korean, and Japanese), the region portion was removed, as it was assumed that publication in these languages would imply samples from Easy Asia already. The Boolean text was thus presented as follows for these searches: (Internet community) AND (Mental health OR Mixed) AND (Behaviours). As the databases employed had different affordances, specific criteria we employed across each platform are specified in Table 2. With all of these procedures completed, 1,110 English papers, 261 Japanese papers, 328 Korean papers, 16 Traditional Chinese papers were retrieved.

**Table 2:** Affordances employed in each database search.

Database	Section/Field	Filters employed
Scopus	Title OR Abstract OR Keywords	Language (English), Journal article or Conference proceeding
EBSCO	Keywords (SU)	Peer reviewed
IEEE	Abstract	N/A
CiNii	Keywords (Free-word search)	Language (Japanese), full-text available, Journal article
KCI	Titles (TI) OR Abstracts (AB) or Keywords (SU)	Journal article, Open access, Regular journal article, Full-text available
Airiti	All text	N/A

“SU” refers to subject, “TI” refers to title, and “AB” refers to abstract in-platform.

### Screening process

The screening process was conducted in four languages—English, Traditional Chinese, Korean, and Japanese—to ensure the inclusion of relevant studies published in diverse linguistic contexts. For each retrieved paper, the title, abstract, and keywords were evaluated against the eligibility criteria. Four researchers independently read each retrieved paper’s abstract in an unblinded, standardized manner. After the first screening process was finished, irrelevant papers were ruled out, and the relevant or ambivalent papers were saved and entered the second screening by the first author. In other words, every non-English relevant paper was reviewed two times by different researchers. The English relevant paper went through the screening process by the first author only. The ambivalent papers, articles lacking consensus were retained for full-text analysis in second screening until a final inclusion decision was reached. For this second screening, two authors initially reviewed eight English-language articles–roughly 20% of the articles that were available in a language that all authors understood–and achieved a Cohen’s kappa of 0.35, which was insufficient. The two authors met and discussed discrepancies, deciding together on a final screening. Another eight articles were thus randomly chosen in the English pool and a Cohen’s kappa of 0.63 was achieved, indicating acceptable levels of agreement between coders. The other two authors then reviewed the same articles and a Cohen’s kappa of 0.61 and 0.60 were, respectively, achieved. After this, authors worked independently to screen and evaluate the rest of the papers in their respective languages.

The established inclusion criteria required papers to meet two main conditions. First, the papers had to show the intersection of mental health and online support. This could be indicated by having either of these two terms as keywords in the list of keywords, the title, or the abstract. Mental health was considered both in its positive and negative terms here: having a pathology or being at risk for a pathology, or psychological and wellbeing optimization. However, ‘online support’ is a broad term; in the absence of a clear definition, the authorial team thus brainstormed collectively all of the different ways a person could find support using the internet. These included professional avenues like online therapy, informal support through chatting with friends or strangers online, or even passive consumption of mental health content. The complete list of keywords related to mental health *and* online support are presented in Appendix A. Second, the article should be written in English, Traditional Chinese, Korean, or Japanese. In addition to this, English-language papers were required to include at least one term related to the region domain (e.g., “Asia,” “Korea,” or similar expressions) in the title, abstract, or keywords.

Exclusion criteria were applied sequentially; once an article met the first exclusion criterion, it was excluded without further assessment against subsequent criteria. Specifically, studies lacking full-text availability or not addressing mental health online interactions in the regional contexts specified were excluded. Thus, 54 papers — 32 in English, 6 in Japanese, and 16^1^ in Korean — remained after the screening. It is worth noting that none of the papers written in Traditional Chinese met our inclusion criteria, suggesting a major dearth of mental health research in regions using this script (e.g., Taiwan, Hong Kong, Macau, and their diasporas). The full PRISMA process is presented in Figure 1.

**Figure 1:**
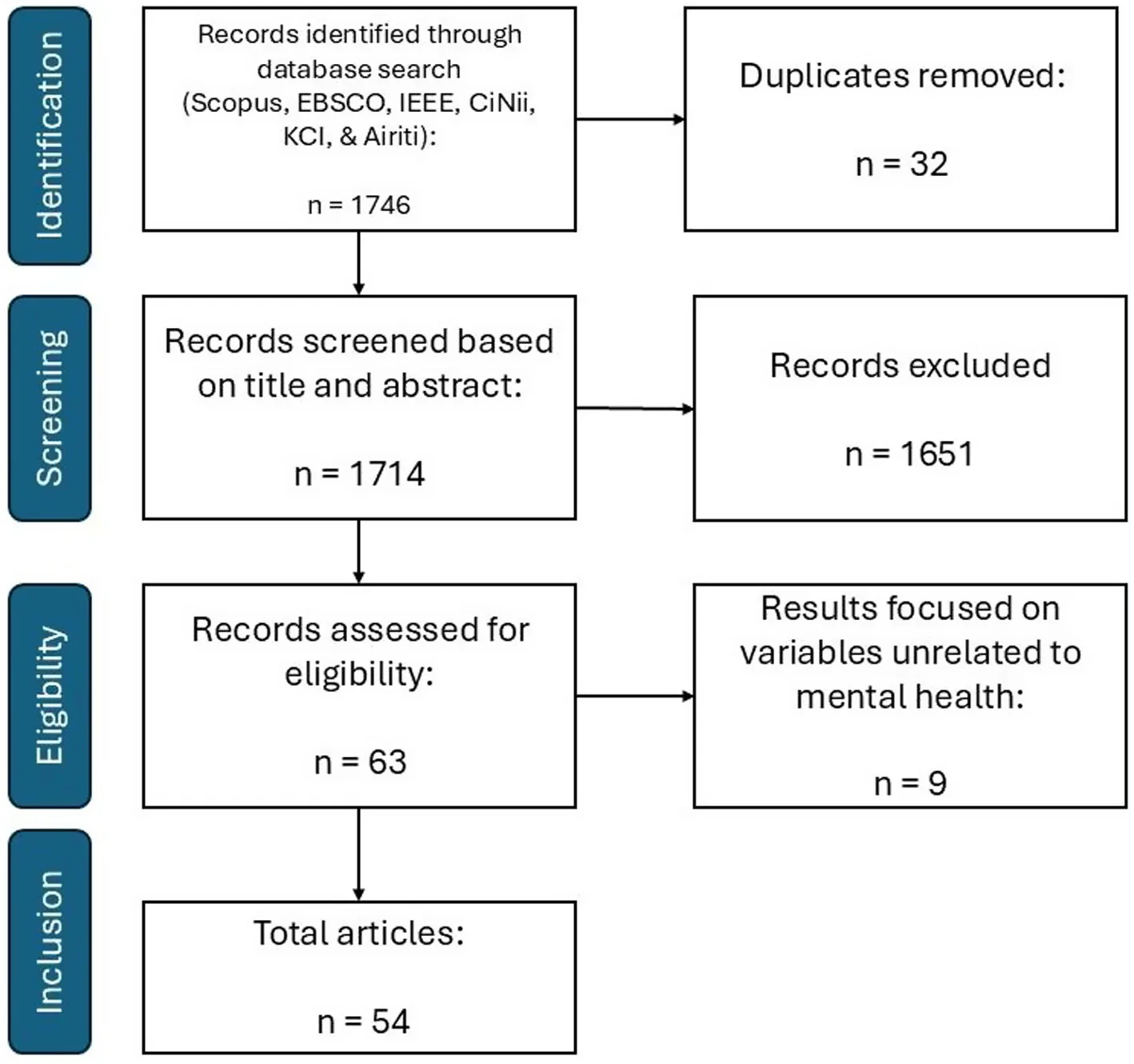
PRISMA flowchart.

### Materials

To assess the methodological quality of the included studies, we used the Mixed Methods Appraisal Tool (MMAT) version 2018 (12). MMAT was chosen due to its wide applicability across qualitative, quantitative, and mixed-methods studies. As the current review included studies employing diverse methodologies, MMAT was considered the most suitable tool for methodological quality assessment. Two researchers independently evaluated the Korean and Japanese articles, and other researchers assessed the English articles. No Traditional Chinese articles were evaluated because none remained after the screening stage. In accordance with MMAT guidelines (12), we did not calculate overall scores, instead, we reported the ratings of each criterion for each study (see Appendix C). The potential influence of study quality on the interpretation is discussed. Furthermore, studies with lower methodological quality were not excluded from the analysis based on the MMAT guidelines; however, these studies are mentioned where relevant in the results. A complete evaluation using the MMAT of for each article included in the study can be found in Appendix C.

### Analytical strategy

Given the relatively small number of articles gathered, a qualitative, grounded theory (92) approach was undertaken to identify patterns within the articles collected. Each author corresponding to their language, with the author specializing in Traditional Chinese assisting the author specializing in English due to the discrepancy in articles between these two languages, reviewed each article and identified themes present in the article. These were in part derived from our ample keyword database we constructed at the beginning; however, authors were also free to identify and list themes that emerged from the dataset, as this is the basis of grounded theory. Themes included topics of interest, keywords, methodologies, and findings. Those identified from the original keywords and those discovered independently by our team through reading the texts were then compiled into a broader list of themes, with overlapping and language-distinct themes being identified through collective review of the completed list as a team.

## Results

For a complete summary of all studies and their included themes, as well as their regions of origin and language of publication, please refer to Appendix B.

### Regional patterns

Upon review, the majority of our articles (18 from Mainland China, 4 from Taiwan, and 3 from Hong Kong) used Chinese-speaking samples, followed by South Korean (20), then Japanese (5). The remaining articles either sampled a variety of countries (3), or performed a direct cultural comparison (1). Within these regions, specific methodological and topical themes emerged, some stronger than others.

#### Mainland China

Within articles focused on Mainland China, one theme dominated above all others: a concern for internet gaming disorder (IGD), occasionally called internet gaming addiction (see Appendix B). Of the 18 articles based in this region, 14 had the primary purpose of exploring this diagnosis, either through discovering risk and/or protective factors [e.g., (13, 14)] or by developing prevalence rates [e.g., (15)], sometimes in tandem (e.g., Yang X. et al., 2020). The remaining four articles focused on disparate topics, one of which framed internet usage–not social media usage–as a potential avenue for mental health improvement (16), another which was neutral in its stance on online presence and mental health (17), one which framed it negatively (18), and a final one which mentioned social media’s usefulness as an information channel (19).

Methodologically, surveys dominated, with one article performing text/sentiment analysis (16), another performing an experiment (13), and a final article performing discourse analysis (17); one survey study did, however, also include interviews (20). Notably, two of the articles that were methodologically diverse were also part of the three articles that were *not* focused on IGD (16, 17). Generally, articles focusing on this region have a notable preference for a quantitative and/or cross-sectional approach, and are–interestingly–published almost exclusively in English (see (21) for an exception published in Japanese). In addition, there is a major negative framing of any usage of online resources across these articles. With only a few exceptions [see (16, 17, 19)], articles in this region frame internet usage as either an addiction at worst, or something to be feared and avoided at best. This leaves ample gaps in research available to those interested in studying this region, both methodologically and practically. For instance, those who are using online resources positively are thus far largely untouched academically; these people could be interviewed solo or in focus groups to understand their experiences and generate new variables to examine *positive* online experiences and usage that we have seen in other regions [e.g., (22)].

Modern China is a research powerhouse, with recent statistics suggesting that it outperforms most other countries in terms of publishing in high-impact journals (23). To do this, it seems to have largely followed the early American format of investing in research through universities, with a particular emphasis on technology development and refinement (23). In terms of research tradition, this seems to have led to a focus on quantitative, large-scale works within the world of social sciences: articles that would be competitive on the international stage. The enormous population coupled with this increase in funding and trained personnel allows for the quantity and quality (see Table 3c in Appendix C) of publications we found from this region.

#### Taiwan and Hong Kong

In the other Chinese-speaking regions that emerged in our dataset, the focus on IGD remained, with five out of the seven articles looking at this diagnosis (24–28). The two that did not examine IGD, however, focused on internet dependence more broadly (29) and the use of social media during times of political crisis (30). Where these regions differed from Mainland China was in relation to the skew of the ages targeted. While Mainland Chinese articles tended to focus on younger samples, articles in Taiwan and Hong Kong usually focused on either adolescents (25–27) or adults (24, 28–30). Surveys remained the most popular choice (24, 25, 27–30), with only one study using interviews (26).

Like in Mainland China, articles based in Taiwan and Hong Kong heavily favor the quantitative, cross-sectional approach. These are also all published in English; despite these two regions using traditional Chinese characters, we were unable to find any articles published in Mandarin on subjects related to mental health and online behavior. There are, therefore, similar methodological and practical gaps that researchers still have room to fill in this space. Positive elements of gaming and internet usage remain largely unexplored, and the potential of online alternatives to traditional talk therapy or support groups are yet to be acknowledged here. This is of particular interest given the face culture of these regions [see (31)]; when it may cause embarrassment or shame to seek mental help in person, the anonymity or pseudonymity of an online space could potentially provide an alternative space in which to heal.

Sharing China’s Confucian background, Taiwan and Hong Kong both favor similar research topics and techniques; this may even be enhanced by their historic and current ties to major English-speaking countries like the United States and the United Kingdom. However, lacking the same amount of funding and sheer population, Hong Kong and Taiwan are unable to produce the number of articles that China does. The quality, however, does not suffer, as evidenced in MMAT scores (see Table 3c of Appendix C); what *is* produced is handled carefully.

#### South Korea

In fact, it was in South Korea that we found our first studies into online support groups (32, 33). The first of these looked at how women with depression can use social media to express their symptoms and shape their own narratives, having a therapeutic effect on symptoms (32). The second compared online support sides for suicide with their dark counterparts–those advocating for suicide–and discussed how online discourse, even with the best intentions, can still be liable for the offline actions of their users (33). In both of these articles, online support is framed as something amateur and informal rather than a more ‘legitimate’ alternative or supplement to offline therapy or social support.

Moving from these two examples, we still found a considerable focus on gaming, but less so on the IGD diagnosis specifically. In contrast, Korean articles framed gaming as an activity that could be adaptive (34) or maladaptive [e.g., (35)] depending on circumstances and usage patterns. Only three articles emphasized IGD as a diagnosis (34, 36, 37). When speaking of addiction, Korean articles tend to speak of internet [e.g., (38)], social media [e.g., (39)] or smartphone addiction [e.g., (40)], irrespective of what exactly the participants are doing when using these resources; even then, the terms ‘dependence’ or ‘overuse’ are often preferred over ‘addiction’. Many Korean articles also focused on the intersection of social media usage and depression among a variety of age groups from adolescents to senior citizens [e.g., (38, 41, 42)], while others focused specifically on the experiences of women regarding sexual violence and/or bodily image [e.g., (43, 44)]. While we still see an emphasis on one’s online life being a threat to one’s offline life via dependence or overuse, Korean articles *do* also present the opportunities of online spaces for mental wellbeing improvement.

Methodologically, we see considerable diversity in South Korea. Though surveys remained the dominant paradigm (twelve), we also found two longitudinal panel studies (38, 44), an experimental intervention (37), a mixed-methods study (33) and four content analyses (32, 42, 45, 46), many of which used a combination of machine learning and text analysis. Quantitative work is still the norm, but we can see breaks from the cross-sectional preference through the longitudinal designs and the content analyses, two of which feature computational models designed to predict or assess mental health trends in the country using social media posts (42, 46). It is also worth noting that of our 20 Korean articles, only 4 were written and published in English, suggesting a comparatively robust local publishing culture that is open to mental health topics. Still, qualitative works are lacking, leaving additional room for new and existing scholars to explore the intersection of mental health and online spaces in South Korea through interviews, focus groups, ethnography and the like.

South Korea, unlike the previous regions examined, has a unique focus on decolonizing research and building uniquely Korean foundations to the research involving their people [see (47)]. Though they maintain the Confucian backbone that East Asia largely shares, leading to strong governmental support and hierarchical workplaces (see (48) for examples of the impact of these factors), there is a distinct motivation to test assumptions based on Western models, leading to thorough, large-scale research initiatives (see Table 2c in Appendix C).

#### Japan

The Japanese context provided the least number of papers (six), and as such, it is difficult to pinpoint specific themes. Two of the papers focus on the social network LINE: an app that includes instant messaging, news, and a variety of other functions [see (49)]. These two papers focus on high school (50) and first-year university students (51) respectively, and explore the feelings of rejection after being ignored in instant messaging. Nakayama (52) takes a broader view, instead looking at high school students’ friendships and how these can shape their social media usage. Two other articles focus on flaming (53) and defamation (54) on YouTube, examining how to detect flaming and how defamation spreads, respectively. The final article is theoretical, reframing the *hikikomori* phenomenon–in which people choose prolonged social withdrawal–as an international phenomenon that has changed markedly after the new communication technologies that emerged during the COVID-19 pandemic era became normalized (55).

In terms of methodology, we see a distinct departure from the survey paradigm present in the other regions; in fact, only *one* of our Japanese sample studies involved surveys (52). Instead, Japanese scholars preferred papers focused on theory-crafting (50, 55), interviews (51), or content analyses (53, 54). Contrary to other parts of East Asia, there appears to be a distinct preference for qualitative or critical work in terms of Japanese publications. Like in South Korea’s case, the majority of our Japanese samples also came from articles written in Japanese; only one paper was written and published in English (55). Also like in the rest of East Asia, there is an overarching negative framing around online behavior, with only one article (55) mentioning positive outcomes of participating in online culture and discourse. Given the general dearth of work on Japanese samples in the mental health and internet intersectional space, there is ample room for scholars of all traditions to continue researching these issues and potential benefits in the future.

As for why this may be the case, Japan is arguably the most isolated in terms of academia [see (56–58)]. While its Asian neighbours have plenty of English-language programs available for students and hire foreign faculty somewhat regularly, Japanese universities continue to operate almost exclusively in Japanese (58) while foreigners who come exist in a kind of linguistic enclave leading to feelings of tokenisation (56, 57). This exclusionary environment could explain why there is such a different methodological focus and a heavier emphasis on publications in the local language.

### Global and comparative work

Perhaps unsurprisingly, all four of the global comparison articles are available in English (see Appendix B). There were few defining threads in terms of comparative work; each article was distinct. The first was a text-mining study of COVID-19 tweets internationally, finding that public social media posts are a useful way to keep tabs on evolving mental health crises like loneliness (59). The second was a focus-group study involving 13 different countries (China and Indonesia representing Asia) in which adolescents explained their ambivalence toward digital communication and mental health, saying it was useful for building up one’s network, but came with significant risks in regards to cyberbullying, cancelling, etc. (60). The third global article was a scoping review of mental health apps which determined that available therapy apps based on existing offline therapeutical techniques such as meditation, breathing exercises, and cognitive behavior therapy (CBT) could be effective for stress, anxiety and depression; some even increased general well-being (61). The final study was a survey-based comparison between Japanese and Chinese young adults who had IGD tendencies (or diagnoses) which found that while Japanese youth tended to suffer more from IGD tendencies and psychological distress, mindfulness could help everyone–especially women–to mitigate these effects (62).

Though these studies are methodologically and theoretically diverse, the fact that only four emerged in our study is surprising. Whether consciously or unconsciously, East Asian regions are, for the most part, either being left out of the global conversation around mental health and online support options, or intentionally opting out. As systematic reviews are descriptive, we cannot say which is the case; what we *can* say is that we as scholars have the capacity to change this through increased international collaboration and support for scholars publishing in these fields. Our results would suggest that there is a need for increased work in this physical and disciplinary space.

### Broader themes of research

This subsection will focus on the findings related to these themes across regions. Methodologies will also be touched upon where relevant.

#### Internet gaming disorder/addiction (IGD/A)

Of the 21 articles that fell under this broader theme, 17 of these used Chinese-speaking samples (all mainland, with the exception of (24), who was Taiwan-based) and were published in English, while 4 focused on Korean samples. Important to note in this particular theme is the discourse around IGD and the diversity of diagnostic criteria [see (63)]; we will therefore first specify which articles used which criteria and how before entering into the more specific themes. As Cheng et al.’ (13) study focused on early intervention in childhood, they did not use a diagnosed population and did not specify any IGD diagnostic criteria in their study; Lee and Jeong (35) similarly only mention IGD, and focus on ‘problematic gaming’ as opposed to a specific diagnostic criterion. Wan and Chiou’s (26) study predates the addition of IGD to diagnostic manuals, and so it relied on the authors’ subjective assessments as clinicians. Like Cheng et al.’ (13) work, Tsui and Cheng’s (25) focused on risk for the *development* of IGD, and therefore did not include specific IGD criteria in their work.

The rest of the articles relied on scales developed based on the 5th edition of the Diagnostic and Statistical Manual of Mental Disorder’s (DSM-5) diagnostic criteria for IGD diagnosis (14, 15, 27, 28, 62, 64–71) or on clinical interviews to assess IGD symptoms based on the DSM-5’s diagnostic criteria (20, 24). The one exception to this is Shim’s (37) piece, which had no specification of diagnostic criteria. According to the DSM-5 (72), a person needs to meet five of the following nine criteria in order to be diagnosed as addicted to gaming: they need to be preoccupied with gaming, where gaming dominates their inner lives and activities; experience symptoms of withdrawal when gaming is restricted; develop a tolerance to gaming, requiring more to achieve satisfaction; have failed to stop or control their gaming in the past; have lost interest in other activities; continued playing despite knowing the negative impact it is having on them; lie to others about how much they are gaming; use gaming as a form of escapism; or have lost friends or employment due to gaming. All of the scales in the studies specified used self-report instruments that had been validated by other scholars in Chinese or Korean that asked participants to rate themselves on these nine criteria, allowing for a kind of proxy diagnosis of participants.

##### Risk and protective factors

Of the 21 articles in the broad category of IGD, 16 discussed risk and protective factors for the development of IGD, making it the most popular sub-theme within the IGD category. Many articles reported prevalence statistics of IGD in their samples, which ranged anywhere from 11.60 to 17.00% (15, 34, 66, 69, 70). However, the core focus of these articles was on (a) what creates a risk of developing IGD, and (b) which factors could prevent or mitigate its impact. In terms of risk factors, several were identified: stress [e.g., (64)], existential emptiness (37), personality traits [e.g., (66)], playing games as a form of escapism [e.g., (36)] or to emotionally cope (e.g., (26)], having already been diagnosed with generalized anxiety disorder (20), and being surrounded by others who game regularly [e.g., (27)]. Protective factors include parental understanding of games and internet culture and risks [e.g., (69)], a sense of hope (68), the presence of a strong offline social network (35), psychological resilience [e.g., (25)], and cognitive confidence (71). Generally, articles in this category were descriptive in nature–usually employing surveys or interviews–and their primary goal was identifying key variables for future, more specific work.

##### Consequences

Five articles, sometimes in describing the protective and risk factors, sometimes independently, also noted several consequences of being a person with IGD. The major symptom found across several studies was that having IGD can actually increase the stress that many people game to avoid [e.g., (24, 64)]. The same has been found true for depressive and anxious symptomology [e.g., (25, 35, 68)]. For those diagnosed, the relaxing and coping typically associated with gaming online, alone or with others, become paradoxically addictive according to the studies in our dataset. Like in the case of the risk and protective factors, studies in this theme tended to be largely descriptive with findings focused on things like prevalence rates as opposed to phenomenology.

##### Interventions and therapy

Finally, two articles talked about potential interventions for those who have already been diagnosed (13, 37). The first is designed for children and was tested in China (13). Focusing on what they call the P-A-G-E framework–psychological resilience, active coping, growth mindset, and emotion regulation–they had success in reducing disorder symptoms and negative affect. Shim’s (37) study takes a spiritual approach and focuses on adults. According to Shim (37), those experiencing IGD are also experiencing an ‘existential emptiness’ that they are trying to fill with games. By addressing this core need in religious counseling, Shim (37) argues, IGD symptoms and behaviors can be reduced. Both articles had the core finding that intervention in IGD cases can be successful–either pre- or post-diagnosis (13, 37).

##### Social media, internet, and smartphones as addictions and/or dependencies

Of the 10 articles focusing on alternative addictions/dependencies (social media, internet, or smartphones), eight came from South Korean teams (38–41, 65, 73–75); the other two articles came from Taiwan (29) and mainland China (64). Like in the case of IGD literature, articles in our dataset focusing on internet, smartphone, or social media dependencies also explored risk factors and consequences, with a lack of social connection emerging as a major predictor across regions for social media, smartphone, and internet dependency (29, 38, 41). Interestingly, however, no therapies or interventions were covered with any depth; the focus in these articles was much more descriptive than proscriptive. For instance, Huang (29) found that when faced with crises of either intimacy or identity, having a higher dependence on the internet led to either more difficulty or a total inability to solve these crises. Gu and Mao (64) in their study of university students found that academic stress, though less severe than an intimacy crisis, can also trigger problematic smartphone use and internet dependency. Oh and Kim (38) add additional context to this finding, stating that an important antecedent of internet dependence is a lack of social support offline. In their study of South Koreans over 65, Kang and Lee (41) found that depression completely mediates the relationship between life satisfaction and internet addiction. Together, these articles suggest that those who are dependent on the internet [or internet access via smartphone, as described by (40, 75)] are often that way because they are seeking something they lack offscreen, which can lead to profound emotional distress. Unfortunately, there is currently a lack of studies examining interventions in these same cases to alleviate that distress.

#### Clinical diagnoses and novel detection methodologies

On a more positive note, research on how to use social media and the internet more broadly for the improvement of human wellbeing appears to be emerging in East Asia as well, although only six were identified in the present work (42, 46, 50, 55, 59). Notably, this broad theme is *not* dominated by a specific region, as almost all regions represented linguistically in this review were encompassed here. Three studies in our dataset indicated that posts on social media can be used to keep track of mental health crises as they evolve (59), detect and remove flaming before it does any damage to other users of social networking platforms (53), and even detect depression trends in the population with an over 80% accuracy rate (46). These two articles both use new machine learning algorithms to flag user generated content, either to remove it in the case of Sakai et al.’ (53) work, or to document and describe it, in the case of Park et al.’ (46) article. Japanese theorists have also posited potential mechanisms for online toxicity (50) and how *hikikomori* can potentially use new communication technologies to broaden their horizons (55). Kim et al. (42) even report that incidents of posting about self-harm are decreasing gradually since the COVID-19 pandemic. Although only a small number of articles, particularly compared with the overwhelming number of negative-framing articles surrounding mental health and online presence in East Asia, they are the most regionally and methodologically diverse, suggesting an avenue for innovation in this area in the coming future.

#### Body and identity

This final, broad category of 12 articles captures three subthemes: (a) gender-based studies (focusing on women), (b) self-image/concept, and (c) friendships and social media. All of these relate in some capacity to either one’s body and bodily autonomy or to one’s social identity. In terms of regional distribution, this broad theme, too, was covered by all of the regions included in the present review. Methodologically, we find some diversity–content analyses and interviews feature regularly–but surveys still dominate as the primary mode of investigation.

In the first subtheme of gender (four articles), we see women negotiating their identity in online spaces in ways that they would not be able to do publicly offline (17, 32). In Chang’s (32) content analysis of young Korean women’s social media posts, she found that they were using the platforms as a kind of simultaneous diary and soapbox in which they freely vented about their depressive symptoms. Ren and Yao’s (17) content analysis of millennial women on Chinese social media observed that they will jokingly refer to themselves as ‘old’ as a kind of bonding practice and inside joke, despite being in their 20’s and 30’s. In both articles, the pseudonymity of online fora allows for the expression of the ‘ugly’ parts of ourselves that face culture typically suppresses in favor of a perfectionistic self-presentation. One article focusing also found that women in particular suffer inordinately from online sexual violence, and that this can dramatically affect their fear response offline toward sexual crime (43), while Zhang (21) found that women gamers also deal with microaggressions and more overt harassment based purely on gender in online gaming environments. Articles in this subtheme together convey the message that the internet is not an equal place for women, but that there is the potential for unique self-expression that local cultures can sometimes make challenging.

For the second subtheme, we found four articles (18, 44, 60, 76). Articles focusing on self-concept and/or self-image find that excessive passive use of social media like TikTok is associated with lower appearance and weight esteem (18), and that upward social comparison is critical to getting benefits out of social media usage [e.g., (76)]. In essence, their findings suggest that we must master social media in order to enjoy it without damaging our self-esteem: being active, mindful consumers who seek out ways to feel good about ourselves through others’ content. It should be noted, however, that these articles are largely descriptive in nature. Yang et al.’ (44) article takes a different approach to bodily autonomy, focusing on sexual online experiences in early childhood. They found that those who are vulnerable offline–experiencing anxiety and/or depression–are also more vulnerable to unwanted sexual interactions online. Similarly, Ziegel et al. (60) conducted 71 focus groups in 13 countries across four continents about mental health in adolescence and found that social media was critical in “engendering social comparison, promoting gender stereotypes, [and] increasing exposure to bullying and harassment” (p. 405). Overall, the research focuses on the dangers of social media and internet access, and suggests that reaping rewards from social media engagement requires consistent discernment and vigilance.

For friendship, we identified four more articles–one published in Korean (45), two in Japanese (51, 52), and one in English by a team out of Hong Kong (30). We shift the focus toward social media specifically with instant messaging capacities, often LINE. In their 2017 article, Zhu et al. (30) describe how during the Umbrella Movement protests in Hong Kong, their participants actively engaged privacy affordances to limit their exposure to the political crisis, even going so far as to unfriend and block those who posted excessively about this topic. On the opposite end of the interpersonal spectrum, Tanemura and Tsuyoshi’s (51) interviews with first-year university students suggest that messages on social media being ignored triggers an isolation anxiety effect, making students fear ostracism. Indeed, Nakayama (52) finds that, among high school students, the kinds of friendships they have directly impacts how and how often these students even *use* social media to begin with. All of these articles demonstrate how anxiety can be triggered and managed using social media, and also how friendships are navigated in a digital age where fear is omnipresent.

### Uncategorized articles

Five articles identified were about mental health and online communication, but did not cleanly fit into any of the broader themes identified earlier. The first of these, by Du et al. (19), employed a survey to explore how different types of workers across different fields in mainland China experienced the COVID-19 pandemic. They found that those in the medical field were most vulnerable to depression and anxiety during this time, and that women were especially prone. Like the majority of articles in this review, this one is descriptive in nature. The second article used a content analysis of Weibo posts to explore how people can more effectively use social media to cope with stress (16). Their findings suggest that people can engage in what they call “mental time travel” combined with meaning-making–referring to identifying and reflecting on the causes and effects of moments in life–can allow people to experience positive emotions even while stressed. This, like the novel detection theme articles, suggests there is space for positive framing of online connection in this part of the world.

The third article, by Ueda and Takanashi (54), uses a novel AI-based sentiment analysis to study echo chambers in cases of online defamation. They found that stronger negative emotions, especially anger, were predictive of engaging in defamation behavior. As their focus was on the perpetrators and not the victims, this has more protective implications for mental health as opposed to intervention ideas for those struggling; however, it still spoke of the anxiety and depression symptoms experienced by those who were harmed by the defamation. A fourth study by Kim et al. (42) used a text-mining approach with topic modelling to identify self-harm trends among Korean adolescents by web-scraping social media posts. Their finding was that, based on social media posts, self-harm has increased among adolescents in South Korea, particularly in the post-pandemic era. Finally, also out of Korea, Moon (33) conducted a case study of websites either promoting or denouncing suicide, finding that both sites brought significant risk to users, despite their opposing intentions. Although these articles stood alone thematically, they still bring insight to the kinds of topics of interest in this part of the world, as well as shine a light on mental health issues being experienced and documented.

## Discussion

The present study aimed to systematically review the state of literature at the intersection of mental health and the internet in the three East Asian regions. To that end, a broad, interdisciplinary data collection was undertaken and rigorously reviewed, resulting in 54 unique articles. Novelly, this review purposely did *not* exclude articles written in languages other than English, but actively *sought* articles from other local language groups to the best of the authorial team’s language capacities. Our review suggests that, while there is hope for research examining the potential of social media and internet resources for wellbeing promotion and mental illness mitigation and/or prevention, the current research in this space paints being online as a dark, risky undertaking, particularly for the three East Asian regions’ youth. Research in this part of the world focuses intensely on addiction with comparatively few published studies dealing with non-dependence-related topics. Even when dependence or addiction are *not* the key variable(s), there is a strong emphasis on the consequences of social media consumption as opposed to any benefits that may exist.

This leads to an interesting chicken-and-egg question, especially when it comes to gaming–a key concern in much of the literature reviewed: are the strict policies we often see in East Asia when it comes to online content consumption (e.g., China’s limitations of kids’ gaming time and Thailand’s pornography bans) the result of this research, or is this research inspired as a post-hoc evaluation of these strict policies [see (77, 78)]? In either case, the negative framing around being online could be preventing some of the more positive results we have seen in other research that suggests online support can have mental health benefits [e.g., (79, 80)]. Individually, this stigmatization could lead to people in this region not adopting mental health services–free or paid, professional or informal–if they are only available online. Mental health practitioners too may be facing challenges to not just treat patients or counsel clients, but also convince them that doing so online is a valid option. This stigmatization via negative framing may even be having negative consequences for mental health outcomes at a societal level, as indicated by recent statistics suggesting that depression and anxiety are major contributors in health burden in Asia (81). From a policy standpoint, it could mean that policies that are intended to protect from harm are actually preventing access to healing.

Even in articles written using East Asian samples from a European or North American perspective tend to share this negative framing. These studies are also notably few, particularly given the sheer population (5) and connectivity of East Asia [see (82)]. Given the broad strokes of our database search (see Appendix A), spanning over 25 years of scholarship across Communication, Psychology, Gender Studies, Religious Studies, and others, the fact that so few articles overall have tackled the intersection of mental health and online behavior demonstrates the need for additional research in this space. What we have yet to find out far outweighs what we already know, and so we call global researchers to act: researching these topics in their proverbial backyards for those of us in East Asia, and for those coming from heavily-researched regions, building stronger collaborative networks in this fecund academic ground.

### Research implications

The first and arguably most important implication of this work is methodological: when we conduct systematic reviews in a single language, we are missing a wealth of information. Many of the insights presented in this piece are only possible to uncover and present because we went beyond the English language to seek out local insights. It may be obvious in a case like this one, where region was a factor of particular importance, but the fact remains that English is not the only publishing language. If we purposely exclude all articles that are not published in English, then we are excluding valid academic findings, and severely limiting our systematic reviews. Wherever possible, we encourage scholars to engage with their home language’s scholarship, and if a native speaker of English, seek out collaborations to unlock those academic insights for you and your readers.

The second major implication is that connecting mental health and internet usage positively is still heavily under-researched in the three East Asian regions specified in the present work. The remaining question is *why* that is the case. As in all research, funding could be playing an important role. What gets funded is likely to attract scholars, and it is much easier to create urgency with a perceived crisis than with a perceived opportunity. In the current research climate globally, funding is being regularly cut, leading to many researchers vying for an increasingly small pool of resources (83). Researchers thus need to be extremely cautious of both their theoretical *and* societal impact (84), meaning obtaining the funding necessary to complete their research is contingent on researching something they can convince governments is urgent. When examining how many studies in the present review were about internet gaming in Chinese communities and published after 2021 when China’s notorious gametime restriction policy went into effect (78), this possibility seems a particularly strong one.

Another possibility is the impact of local cultures. As described earlier, much of East Asia can be described as ‘face’ cultures, meaning that reputation borders on sacred, and all efforts must be made to maintain or enhance the reputation of all involved in a conversation or event [see (31)]. Mental health is a sensitive subject at the best of times, and it is possible that admitting the average person could benefit from additional social support may go too far toward admitting that a region has a significant problem in their population, though Chen et al. (81) have shown that this is indeed the case. Lemon et al. (85) suggest that Southern and Eastern Asia also tend not to see mental health as being a part of physical health, meaning mental health promotion is not integrated into the broader public health agenda. Existing efforts also often lack cultural sensitivity, meaning rural populations and certain ethnic or religious groups can be unintentionally left out of the few initiatives that *are* available (85). However, we have to take the wealth of IGD literature into account, as this would seem to go against that narrative. It may be that the underlying cultural factors are causing a disparity in funding and directing research toward offline options, but this would require further research to know for certain.

A final possible explanation is a simple lack of interest or human resources to answer these questions. East Asia is, as described earlier, known for its emphasis on educational achievement and, in recent years especially, its focus on technology and engineering [e.g., (86)]. Social sciences are, generally-speaking, neither encouraged by parents [see (87), for a discussion of this impact in the Asian diaspora] nor as well-paid as their pure STEM counterparts [see (88, 89) for examples]. These societal and economic factors may explain, alongside the general requirement of face cultures to value reputation [see (93)], why there are fewer researchers focusing on this region in the field. To remedy these issues, we would encourage–where possible and reasonable–the creation of new competitively-compensated local academic positions dedicated to mental wellbeing and innovation. With funding and prestige attached, issues of money and face are likely to be mitigated, allowing for East Asia to be better represented in the global mental health conversation.

Finally, this work has uncovered regional patterns that could possibly be affecting–or have affected–the development of mental health research and interventions in the three East Asian regions. The consistent negative framing around online connectivity–particularly among youth, but not exclusively–could be creating or enforcing stigma around activities that have been shown to be beneficial in other parts of the world. Online gaming seems to be a standout example; where gaming is used as a source of connection in times of crisis in the Western world [see (79, 90)], it is largely vilified in the research catalogued in the present work. Instead of a hobby, it is treated like a disease. Social media use, too, is examined almost exclusively when it is in a state of excess, while research in other regions has shown that it is a valuable source of connection for the retired and differently-abled communities (91). By emphasizing harm in mental health research, the three East Asian regions may unintentionally be inhibiting development of the very tools other regions are using for good in the same space.

### Limitations and future directions

Despite our best efforts, there are still limitations to the present work. Not all databases are publicly accessible, nor do all universities pay for access to all databases; we were therefore limited to the ones presented in Table 1. These databases also focus largely on the social sciences and humanities. Although this topic would be of particular interest in the social sciences and humanities, this does not mean that other disciplines have nothing to add. Future work could tap into additional university networks to access more research. This would be especially important for accessing articles published in simplified Chinese characters, something the present team was unable to do due to access and language limitations. Important databases to consider for future work in this space include PubMed for more policy and hard science articles, as well as CNKI and Wanfang for articles in simplified Chinese. With a larger team and more languages involved, more scholarly ground could have been covered, something we hope to see in future reviews on this topic. In its current form, this systematic review can only be generalized to the disciplines and regions covered, which although many, are not all. Even within these, however, we are unable to access articles that have not been indexed; given the existing cultural pressures previously discussed on mental health research in the three East Asian regions, it may be that articles on this topic are not indexed in these high-profile international databases. Future work should aim for even more local-language databases and alternative searching strategies that are *not* database-dependent to reach under-promoted work in this space that may exist.

The zero-yield of Traditional Chinese literature stems from two technical barriers rather than a lack of regional research. First, database architecture limitations prevent local systems (e.g., Airiti, Taiwan Periodical Literature System) from processing the complex, lengthy Boolean strings required by international PRISMA protocols, leading to system collapse and high exclusion rates. Second, bilingual metadata deficiencies in international databases (Scopus, EBSCO) create a “technical blinding” effect. While these databases support multilingual searches, local journals frequently omit or misformat Chinese metadata during English-centric indexing, rendering rich local scholarship unretrievable via localized terms. To overcome these methodological barriers, future systematic reviews should focus on targeted, specialized local-journal databases rather than relying solely on comprehensive global databases. In other words, researchers should manually pre-screen and select relevant local journals as a preliminary step before conducting the search.

Future research would also benefit from focusing on other regions and including those local languages. Southeast Asia or Africa would be of particular interest given their unique histories with mental health and internet connectivity and their wide linguistic diversity. However, even within the present study’s context of the three East Asian regions, there are targets researchers can focus on reaching in the near future. For instance, much of the research compiled here suggests that what is being published on mental health in this region is heavily biased toward quantitative, particularly survey, work. Moving beyond this particular paradigm into other types of quantitative analyses–this can include more content analyses and similarly data-driven approaches, or even experimental, clinical methods testing intervention effectiveness–is a key directive. Evaluating clinical approaches, especially given the negative framing around being online in this region, is a particularly critical step forward. Finally, we are in need of more research in the positive psychology space when it comes to online engagement. While we know of the dangers, the published research in this part of the world would suggest that we are not capitalizing on the mental optimization and wellness potential of the internet. Only once we have thoroughly researched the other side of the proverbial coin will our picture of the mental health landscape in relation to online connectivity be complete.

## Data Availability

The original contributions presented in the study are included in the article/Supplementary material, further inquiries can be directed to the corresponding author.

## Footnotes

1 One paper, discovered to be written in English during full-text review, did not meet the language criteria. However, we included it because it offered valuable insights into depression trends on Korean social media and was published in a Korean journal. More importantly, this paper can be retrieved through our database search.
