# Supplementary material for: Journey to the East: a focused review of online mental health research in East Asia
Source: Front Public Health. 2026 Oct 12;14:1841986. doi: 10.3389/fpubh.2026.1841986 (PMC13696504; doi:10.3389/fpubh.2026.1841986)
Supplement: Supplementary file 1 [file Supplementary_file_1.pdf]

Appendix A

Region: (Asia OR Asian OR "East Asia" OR "East Asian" OR "South Korea" OR Korea OR Korean OR China OR Chinese OR Japan OR Japanese OR Taiwan OR Taiwanese OR Mongolia OR Mongolian OR "Hong Kong" OR Cantonese OR Mandarin)

Internet community: (Discord OR Snapchat OR OnlyFans OR Patreon OR gamer OR gamers OR Twitch.tv OR Facebook OR Instagram OR IG OR FB OR TikTok OR YouTube OR Reddit OR KakaoTalk OR X OR Twitter OR LINE OR WeChat OR Weibo OR Bilibili OR Xiaohongshu OR Whatsapp OR PTT OR Douyin OR Dcard)

Mental health: ("mental health" OR wellbeing OR depression OR depressed OR anxiety OR anxious OR "social anxiety" OR "socially anxious" OR vulnerability OR resilience OR inclusivity OR addiction OR suicide OR "suicidal ideation" OR "self-help" OR therapy OR therapist OR "group therapy" OR "body image" OR stigma OR "self-concept" OR identity OR stress OR distress)

Behaviours: (toxicity OR toxic OR troll OR trolling OR "hate speech" OR polarization OR "online polarization" OR coping OR "coping strategies" OR "coping techniques" OR "defense mechanisms" OR "peer support" OR "digital therapy" OR "chatbot therapy" OR "AI therapy" OR "digital vigilanteism" OR "digital vigilante" OR flaming OR doxxing OR harassment OR "sexual harassment" OR swatting OR mansplaining OR sexism OR racism OR microaggressions OR ageism OR ableism OR ageist OR ableist OR sexist OR racist OR "social comparison" OR "trash-talking" OR "trash-talk" OR stereotyping OR stereotype OR "peer pressure" OR ostracism)

Mixed: (ehealth OR polarization OR "online polarization" OR "digital therapy" OR "chatbot therapy" OR "AI therapy"

Appendix B

DOI[1]	Language	Year of Publication	Topic(s)	Methodology(ies)
10.2196/63407	English	2025	Blog Posts, Stress	Content Analysis
10.1556/2006.2024.00052	English	2024	Gaming Disorder, Mental Wellness, Child Sample	Experiment
10.1089/cpb.2006.9.762	English	2006	Gaming Disorder, Adolescent Sample	Interviews
10.2196/24240	English	2020	Mental Health, Pandemic, Industry Sample	Survey
10.3389/fpsyt.2022.1032700	English	2023	Academic Stress, Problematic Smartphone Use, Gaming Addiction, Young Adult Sample	Survey
10.1089/cpb.2006.9.571	English	2006	Internet Dependence, Identity, Intimacy, Young Adult Sample	Survey
10.1016/j.jpsychires.2020.11.015	English	2022	Loneliness, Pandemic, X/Twitter	Content Analysis
10.1186/1471-2458-14-426	English	2014	Gaming as Coping, Depression, Worry, Mentorship, Young Adult Sample	Survey
10.3390/ijerph18052397	English	2021	Bullying, Gaming Disorder, Parenting, Child Sample	Survey
10.3389/fpsyt.2020.598585	English	2020	Gaming Disorder, Personality Psychology, Adolescent Sample	Survey
10.3390/ijerph18041771	English	2021	Gaming Disorder, Coping, Stress, Adult Sample	Survey

[1] In the absence of a DOI number, a link to the article is provided.

10.1186/s12888-022-03778-6	English	2022	Gaming Disorder, Depression, Family Support, Peer Support, Young Adult Sample	Survey
10.1192/bji.2024.38	English	2024	Hikikomori, Pandemic, Online Coping	Theory
10.1089/g4h.2020.0228	English	2021	Gaming Disorder, Adolescent Sample	Survey
10.1089/cyber.2022.0169	English	2023	Body Image, TikTok, Social Media Usage	Survey
10.1016/j.jaging.2025.101325	English	2025	Ageism, Social Media, Young Adult Sample	Discourse Analysis
10.3389/fpsyt.2021.722353	English	2021	Gaming Disorder, Risky Behavior, Mental Health, Adolescent Sample	Survey
10.1556/2006.6.2017.088	English	2017	Gaming Disorder, Generalized Anxiety Disorder, Behavioral Inhibition, Young Adult Sample	Survey
10.2196/41793	English	2023	Depression, Internet Use, Social Comparison, Adult Sample	Survey
10.1556/2006.6.2017.007	English	2017	Gaming Disorder, Scale Development, Adult Sample	Survey
10.3390/ijerph19052951	English	2022	Gaming Disorder, Coping Resources, Distress, Cross-Cultural Comparison, Young Adult Sample	Survey
10.1089/cyber.2015.0345	English	2015	Gaming Disorder, Peer Influence, Expectancy Theory, Adolescent Sample	Survey
10.1186/s12888-025-06910-4	English	2025	Gaming Disorder, Stress, Mindset, Young Adult Sample	Survey

10.3390/ijerph20043375	English	2023	Gaming Disorder, Suicidal Ideation, Emotion, Hope, Adult Sample	Survey
10.3390/ijerph19148332	English	2022	Gaming Disorder, Social Cynicism, Motivation, Pandemic, Adult Sample	Survey
10.4306/pi.2014.11.1.24	English	2014	Online Toxicity, Internet Use, Child Sample	Longitudinal Survey
10.3390/ijerph17020579	English	2020	Gaming Disorder, Social Behavior, Adolescent Sample	Survey
10.2196/26810	English	2021	Gaming Disorder, Personality Psychology, Self-Control, Adolescent Sample	Survey
10.1016/j.addbeh.2023.107836	English	2023	Gaming Disorder, Metacognition, Stress, Coping Mechanisms, Young Adult Sample	Survey
10.1080/10584609.2016.1222471	English	2017	Online Toxicity, Political Ideology, Boundary Setting, Young Adult Sample	Survey
10.1016/j.jadohealth.2024.02.037	English	2025	Mental Health, Digital Communication, Online Gaming, Cross-Cultural Comparison, Adolescent Sample	Focus Groups
10.30806/fs.25.2.202006.5	Korean	2020	Gaming Disorder, Therapy, Spirituality/Religion	Intervention
10.33071/ssricb.49.1.202502.115	Korean	2025	Smartphone overuse, Emotional Regulation, Young Adult Sample	Survey
10.23844/kjcp.2024.11.36.4.1709	Korean	2024	Self-Harm, Adolescent Sample, Young Adult Sample, Social Media, COVID-19	Content Analysis

10.35734/karp.2019.26.3.003	Korean	2023	Social Comparison, Social Media, Self-Esteem, Young Adult Sample	Survey
10.3743/KOSIM.2022.39.1.091	English[2]	2022	Depression, Social Media	Content Analysis
10.30719/JKWS.2021.09.37.3.1	Korean	2021	Women, Depression, Online Forums, Political Leanings	Online observation and Interviews
10.15703/kjc.24.1.202302.23	Korean	2023	Time, Social Media Addiction, Avoidance, Young Adult Sample	Survey
https://www.kci.go.kr/kciportal/ci/sereArticleSearch/ciSereArtiView.kci?sereArticleSearchBean.artiId=ART002908602	Korean	2022	Self-Concept, Social Media Addiction, FOMO, Social Comparison, Young Adult Sample	Survey
10.17315/kjhp.2023.28.3.007	Korean	2023	Depression, Anxiety, Internet Dependence, COVID-19, Social Support, Adult Sample	Longitudinal Survey
10.31888/JKGS.2023.43.3.375	Korean	2023	Depression, Internet Addiction, Life Satisfaction, Senior Citizen Sample	Survey
10.33770/JEBD.40.1.10	Korean	2024	Self-Concept, Social Media Addiction, Perfectionism, Social Comparison, Adolescent Sample	Survey
10.35734/karp.2022.29.2.004	Korean	2022	Self-Harm, Social Media, Online Fora, Depression, Isolation, Adolescent Sample, Young Adult Sample, COVID-19	Content Analysis
https://www.kci.go.kr/kciportal/ci/sereArticleSearch/ciSereArtiView.kci?sereArticleSearchBean.artiId=ART001462821	Korean	2004	Suicide, Websites, Counselors/Therapists	Interviews and Content Analysis

[2] This was found on Korean database KCI, despite being written in English.

https://www.kci.go.kr/kciportal/ci/sereArticleSearch/ciSereArtiView.kci?sereArticleSearchBean.artiId=ART002668124	Korean	2020	Online Behavior, Sexual Violence, Social Media, Women, Fear, Adult Sample	Survey
10.20879/kjjcs.2018.62.6.006	Korean	2018	Mental Health Apps, Adult Population	Systematic Review
10.17315/kjhp.2017.22.2.011	Korean	2017	Gaming Motivation, Gaming Addiction, Relationship Satisfaction, Adult Sample	Survey
10.36494/JCAS.2019.12.36.4.41	Korean	2020	Anxiety, Smartphone Usage	Theory
https://kguopac.kanto-gakuin.ac.jp/webopac/catdbl.do?pkey=NI30000676&hidden_return_link=true&startpos=-1	Japanese	2015	Instant Messaging, Ignoring, Anxiety, Young Adult Sample	Interviews
10.20776/s18848818-14-p1	Japanese	2021	Cyberbullying, Instant Messaging, Education	Theory
https://www.jstage.jst.go.jp/article/digrajprocsummer/2023/0/2023_48/_pdf/-char/ja	Japanese	2023	Gender, Online Gaming, Misogyny, Adult Sample	Interviews
10.11425/sst.7.127	Japanese	2018	Friendships, Social Media Use, Adolescent Sample	Survey
https://api.lib.kyushu-u.ac.jp/opac_download_md/4740674/4740674.pdf	Japanese	2021	Flaming, Machine Learning, Content Moderation	Content Analysis
https://edo.repo.nii.ac.jp/records/1069	Japanese	2022	Defamation, YouTube, Artificial Intelligence, Emotions	Content Analysis

Appendix C

Table 1c.

Japanese articles' MMAT scores.

**DOI or URL**	**Year**	**Topics**	**Method**	**Passed Screen**	**Criteria 1**	**Criteria 2**	**Criteria 3**	**Criteria 4**	**Criteria 5**
https://kguopac.kanto-gakuin.ac.jp/webopac/catdbl.do?pkey=NI30000676&hidden_return_link=true&startpos=-1	2015	Instant Messaging, Ignoring, Anxiety, Young Adult Sample	Interviews (Qualitative)	Yes	Yes	Yes	Yes	Yes	Yes
10.20776/s18848818-14-p1	2021	Cyberbullying, Instant Messaging, Education	Theory	No	N/A	N/A	N/A	N/A	N/A
https://www.jstage.jst.go.jp/article/digrajprocsummer/2023/0/2023_48/_pdf/-char/ja	2023	Gender, Online Gaming, Misogyny, Adult Sample	Interviews (Qualitative)	Yes	Yes	Yes	Can't tell	Yes	Yes
10.11425/sst.7.127	2018	Friendships, Social Media Use, Adolescent Sample	Survey（4. Quant-non-randomized	No	Yes	Yes	Yes	No	Yes
https://api.lib.kyushu-u.ac.jp/opac_download_md/4740674/4740674.pdf	2021	Flaming, Machine Learning, Content Moderation	Content Analysis	No	N/A	N/A	N/A	N/A	N/A

https://edo.repo.nii.ac.jp/records/1069	2022	Defamation, YouTube, Artificial Intelligence, Emotions	Content Analysis	Yes	Yes	N/A	Yes	N/A	Yes

Table 2c.

Korean Articles' MMAT scores.

**DOI or URL**	**Year**	**Topics**	**Method**	**Passed Screen**	**Criteria 1**	**Criteria 2**	**Criteria 3**	**Criteria 4**	**Criteria 5**
10.30806/fs.25.2.202006.5	2020	Gaming Disorder, Therapy, Spirituality/Religion	Qualitative (Theory)	Yes	Yes	Yes	Yes	Yes	Yes
10.33071/ssricb.49.1.202502.115	2025	Smartphone overuse, Emotional Regulation, Young Adult Sample	Quantitative Descriptive (Survey)	Yes	Yes	Yes	Yes	Can't tell	Yes
10.23844/kjcp.2024.11.36.4.1709	2024	Self-Harm, Adolescent Sample, Young Adult Sample, Social Media, COVID-19	Quantitative Descriptive (Content Analysis - Text mining)	Yes	Yes	Yes	Yes	Yes	Yes
10.35734/karp.2019.26.3.003	2023	Social Comparison, Social Media, Self-Esteem, Young Adult Sample	Quantitative Descriptive (Survey)	Yes	Yes	Can't tell	Yes	Can't tell	Yes

10.3743/KOSIM.2022.39.1.091	2022	Depression, Social Media	Quantitative Descriptive (Content Analysis - Deep Learning)	Yes	Yes	Yes	Yes	Yes	Yes
10.30719/JKWS.2021.09.37.3.1	2021	Women, Depression, Online Forums, Political Leanings	Qualitative (Online observation and Interviews)	Yes	Yes	Yes	Yes	Yes	Yes
10.15703/kjc.24.1.202302.23	2023	Time, Social Media Addiction, Avoidance, Young Adult Sample	Quantitative Descriptive (Survey)	Yes	Yes	Yes	Yes	Can't tell	Yes
https://www.kci.go.kr/kciportal/ci/sereArticleSearch/ciSereArtiView.kci?sereArticleSearchBean.artiId=ART002908602	2022	Self-Concept, Social Media Addiction, FOMO, Social Comparison, Young Adult Sample	Quantitative Descriptive (Survey)	Yes	Can't tell	No	Yes	Can't tell	Yes
10.17315/kjhp.2023.28.3.007	2023	Depression, Anxiety, Internet Dependence, COVID-19, Social Support, Adult Sample	Quantitative non-randomized studies (Longitudinal Survey)	Yes	Yes	Yes	No	Yes	Yes
10.31888/JKGS.2023.43.3.375	2023	Depression, Social Media Addiction, Life Satisfaction, Senior Citizen Sample	Quantitative non-randomized (Survey)	Yes	No	Yes	Yes	Can't tell	N/A
10.33770/JEBD.40.1.10	2024	Self-Concept, Social Media Addiction, Perfectionism, Social Comparison, Adolescent Sample	Quantitative Descriptive (Survey)	Yes	Yes	No	Yes	Can't tell	Yes

10.35734/karp.2022.29.2.004	2022	COVID-19, Self-Harm, Social Media, Online Fora, Depression, Isolation, Adolescent Sample, Young Adult Sample, COVID-19	Quantitative Descriptive (Content Analysis - Text mining)	Yes	Yes	Yes	Yes	Yes	Yes
https://www.kci.go.kr/kciportal/ci/sereArticleSearch/ciSereArtiView.kci?sereArticleSearchBean.artiId=ART001462821	2004	Suicide, Websites, Counselors/Therapists	Qualitative (Interviews and Content Analysis)	Yes	Yes	Yes	Yes	Yes	Yes
https://www.kci.go.kr/kciportal/ci/sereArticleSearch/ciSereArtiView.kci?sereArticleSearchBean.artiId=ART002668124	2020	Online Behavior, Sexual Violence, Social Media, Women, Fear, Adult Sample	Quantitative Descriptive (Survey)	Yes	Yes	Yes	Yes	Can't tell	Yes
10.20879/kjjcs.2018.62.6.006	2018	Mental Health Apps, Adult Population	Systematic Review<br><br>Quantitative Descriptive (Survey)	Yes	Yes	Yes	Yes	Can't tell	Yes
10.17315/kjhp.2017.22.2.011	2017	Gaming Motivation, Gaming Addiction, Relationship Satisfaction, Adult Sample	Quantitative Descriptive (Survey)	Yes	Yes	Yes	Yes	Can't tell	Yes
10.36494/JCAS.2019.12.36.4.41	2020	Pregnant Women, Anxiety, Smartphone Usage, Sleep, Adult Sample	Qualitative (Theory)	Yes	Yes	Yes	Yes	Yes	Yes

Table 3c.

English Articles' MMAT Scores.

	DOI or URL	Year	Topics	Method	Passed Screen	Criteria 1	Criteria 2	Criteria 3	Criteria 4	Criteria 5
1	https://www.jmir.org/2025/1/e63407	2025	Blog Posts, Stress	Content Analysis	Yes	Yes	Yes	Yes	Yes	Yes
2	https://www.akjournals.com/view/journals/2006/13/3/article-p871.xml	2024	Gaming Disorder, Mental Wellness, Child Sample	Experiment	Yes	Yes	Yes	Yes	Yes	Yes
3	https://journals.sagepub.com/doi/abs/10.1089/cpb.2006.9.762	2006	Gaming Disorder, Adolescent Sample	Interviews	Yes	Yes	Yes	Yes	Yes	Yes
4	https://www.jmir.org/2020/12/e24240	2020	Mental Health, Pandemic, Industry Sample	Survey	Yes	Can't tell	Can't tell	Yes	Can't tell	Yes

5	https://www.frontiersin.org/journals/psychiatry/articles/10.3389/fpsyt.2022.1032700/full	2023	Academic Stress, Problematic Smartphone Use, Gaming Addiction, Young Adult Sample	Survey	Yes	Yes	Yes	Yes	Yes	Yes
6	https://journals.sagepub.com/doi/abs/10.1089/cpb.2006.9.571	2006	Internet Dependence, Identity, Intimacy, Young Adult Sample	Survey	Yes	Yes	Yes	No	Yes	Yes
7	https://www.sciencedirect.com/science/article/pii/S0022395620310748?via%3Dihub	2022	Loneliness, Pandemic, X/Twitter	Content Analysis	Yes	Yes	Yes	N/A	Yes	Yes
8	https://link.springer.com/article/10.1186/1471-2458-14-426	2014	Gaming as Coping, Depression, Worry, Mentorship,	Survey	Yes	Yes	No	Yes	Yes	Yes

			Young Adult Sample							
9	https://www.mdpi.com/1660-4601/18/5/2397	2021	Bullying, Gaming Disorder, Parenting, Child Sample	Survey	Yes	Yes	Yes	Yes	Yes	Yes
10	https://www.frontiersin.org/journals/psychiatry/articles/10.3389/fpsyt.2020.598585/full	2020	Gaming Disorder, Personality Psychology, Adolescent Sample	Survey	Yes	Yes	Yes	Yes	Yes	Yes
11	https://www.mdpi.com/1660-4601/18/4/1771	2021	Gaming Disorder, Coping, Stress, Adult Sample	Survey	Yes	Yes	Can’t tell	Can’t tell	No	Yes

12	https://link.springer.com/article/10.1186/s12888-022-03778-6	2022	Gaming Disorder, Depression, Family Support, Peer Support, Young Adult Sample	Survey	Yes	Yes	Yes	Yes	Yes	Yes
13	https://www.cambridge.org/core/journals/bjpsych-international/article/paradox-of-hikikomori-through-a-transcultural-lens/4D52ADB7B8AE0850A6A27206ECD1BC67	2024	Hikikomori, Pandemic, Online Coping	Theory	No	N/A	N/A	N/A	N/A	N/A
14	https://journals.sagepub.com/doi/10.1089/g4h.2020.0228	2021	Gaming Disorder, Adolescent Sample	Survey	Yes	Yes	Yes	Yes	Yes	Yes
15	https://journals.sagepub.com/doi/abs/10.1089/cyber.2022.0169	2023	Body Image, TikTok, Social Media Usage	Survey	Yes	Yes	Can’t tell	Yes	Can’t tell	Yes

16	https://www.sciencedirect.com/science/article/abs/pii/S0890406525000192?via%3Dihub	2025	Ageism, Social Media, Young Adult Sample	Discourse Analysis	Yes	Yes	Yes	Yes	Yes	Yes
17	https://www.frontiersin.org/journals/psychiatry/articles/10.3389/fpsyt.2021.722353/full	2021	Gaming Disorder, Risky Behavior, Mental Health, Adolescent Sample	Survey	Yes	Yes	Yes	Yes	Yes	Yes
18	https://www.akjournals.com/view/journals/2006/6/4/article-p564.xml	2017	Gaming Disorder, Generalized Anxiety Disorder, Behavioral Inhibition, Young Adult Sample	Survey	Yes	Yes	Yes	Yes	Yes	Yes
19	https://www.jmir.org/2023/1/e41793	2023	Depression, Internet Use, Social	Survey	Yes	Yes	Yes	Yes	Yes	Yes

			Comparison, Adult Sample							
20	https://www.akjournals.com/view/journals/2006/6/1/article-p11.xml	2017	Gaming Disorder, Scale Development, Adult Sample	Survey	Yes	Yes	No	Yes	No	Yes
21	https://www.mdpi.com/1660-4601/19/5/2951	2022	Gaming Disorder, Coping Resources, Distress, Cross-Cultural Comparison, Young Adult Sample	Survey	Yes	Yes	Yes	Yes	Can't tell	Yes
22	https://journals.sagepub.com/doi/abs/10.1089/cyber.2015.0345	2015	Gaming Disorder, Peer Influence, Expectancy Theory, Adolescent Sample	Survey	Yes	Yes	Yes	Yes	Yes	Yes

23	https://link.springer.com/article/10.1186/s12888-025-06910-4	2025	Gaming Disorder, Stress, Mindset, Young Adult Sample	Survey	Yes	Yes	Yes	Yes	Can’t tell	Yes
24	https://www.mdpi.com/1660-4601/20/4/3375	2023	Gaming Disorder, Suicidal Ideation, Emotion, Hope, Adult Sample	Survey	Yes	No	No	Yes	Yes	Yes
25	https://www.mdpi.com/1660-4601/19/14/8332	2022	Gaming Disorder, Social Cynicism, Motivation, Pandemic, Adult Sample	Survey	Yes	Yes	Yes	Yes	Can’t tell	Yes
26	https://psychiatryinvestigation.org/journal/view.php?doi=10.4306/pi.2014.11.1.24	2014	Online Toxicity, Internet Use, Child Sample	Longitudinal Survey	Yes	Yes	Can’t tell	Yes	Yes	Yes

27	https://www.mdpi.com/1660-4601/17/2/579	2020	Gaming Disorder, Social Behavior, Adolescent Sample	Survey	Yes	Yes	Can’t tell	Can’t tell	Can’t tell	Yes
28	https://www.jmir.org/2021/10/e26810	2021	Gaming Disorder, Personality Psychology, Self-Control, Adolescent Sample	Survey	Yes	Yes	Can’t tell	Yes	Yes	Yes
29	https://www.sciencedirect.com/science/article/abs/pii/S0306460323002319?via%3Dihub	2023	Gaming Disorder, Metacognition, Stress, Coping Mechanisms, Young Adult Sample	Survey	Yes	No	Can’t tell	Yes	Yes	Yes

30	https://www.tandfonline.com/doi/full/10.1080/10584609.2016.1222471	2017	Online Toxicity, Political Ideology, Boundary Setting, Young Adult Sample	Survey	Yes	Yes	Yes	Yes	Yes	Yes
31	https://www.jahonline.org/article/S1054-139X(24)00154-X/abstract	2025	Mental Health, Digital Communication, Online Gaming, Cross-Cultural Comparison, Adolescent Sample	Focus Groups	Yes	Yes	Yes	Yes	Yes	Yes
